# The Contribution of Entomological Surveillance to the Control of Chagas Disease in Endemic Regions: An Integrative Literature Review

**DOI:** 10.1111/tmi.70128

**Published:** 2026-04-22

**Authors:** Daniel Rodrigo de Lima Gomes, Gilliard do Nascimento, Pedro Mariano da Silva Rodrigues, Ana Beatriz da Silva, Ysabele Yngrydh Valente Silva, Ekarinny Myrela Brito de Medeiros, Cléber de Mesquita Andrade, Thales Allyrio Araújo de Medeiros Fernandes, José Antonio da Silva Júnior, Ellany Gurgel Cosme do Nascimento

**Affiliations:** ^1^ Universidade do Estado do Rio Grande do Norte\Brasil University of the State of Rio Grande do Norte Mossoró Rio Grande do Norte Brazil

**Keywords:** Chagas disease, entomology, public health, surveillance, vector control

## Abstract

Chagas disease remains a significant public health challenge in various endemic regions of Latin America. The persistence of vector‐borne transmission highlights the complexity of the issue and the limitations of traditional strategies. In this context, entomological surveillance plays a strategic and multifaceted role, not only in the early detection of triatomine bugs but also in guiding control actions tailored to territorial realities. This study aimed to analyse the contributions of entomological surveillance to the control of Chagas disease in endemic regions, considering the integration of traditional approaches, technological innovations, and participatory strategies. An integrative literature review was conducted based on the guiding question: ‘How does entomological surveillance contribute to the control of Chagas disease in different endemic regions?’ This study is an integrative review conducted in the PubMed, EMBASE, BVS and Web of Science databases, with no time restriction, including primary studies in Portuguese, English and Spanish. A total of 484 studies were identified. After screening and application of the eligibility criteria, 79 articles comprised the final sample, which were analysed through thematic categorisation and descriptive synthesis of the findings. Publications in Portuguese, English, and Spanish, with no time restrictions, were classified into six thematic categories: entomological indices and spatial mapping; factors associated with household invasion; vector control strategies; surveillance methods; professionals' perceptions; and challenges to the sustainability of actions. The findings revealed that entomological surveillance significantly contributes to the control of Chagas disease, especially when it integrates classical indicators, technological tools, and community‐based strategies. The role of entomological indices, interventions in peridomestic ecotopes, and the training of field agents stands out as essential pillars for the effectiveness of interventions. However, vector resistance to insecticides remains a critical obstacle, reinforcing the need for multisectoral, localised and sustainable actions.

## 1 Introduction

Chagas disease, caused by the protozoan *Trypanosoma cruzi* and primarily transmitted by insect vectors of the *Triatominae* subfamily, remains one of the most pressing public health challenges in endemic areas of Latin America. Although vector control interventions have achieved significant progress over the past decades, the persistence of transmission in different socio‐geographical contexts highlights the complexity of the problem and the need for sustainable and context‐specific strategies [1].

In this scenario, entomological surveillance is a fundamental pillar for interrupting the disease's transmission chain. It encompasses early detection of triatomine presence as well as corrective measures such as insecticide application, housing improvements and community mobilisation [2]. However, the effectiveness of these actions depends on the continuity of surveillance activities and the ability of local programs to adapt to the ecological, social, and operational specificities of each territory [3].

While the absence of reported cases in certain areas may suggest effective control, the so‐called ‘epidemiological silence’ often reveals structural gaps in surveillance systems, including low coverage, weak communication channels between health services and communities, and limited response capacity to reinfestation [4].

Given the challenges posed by the complexity of peridomestic ecotopes—such as chicken coops, corrals, woodpiles, and the presence of synanthropic animals—recent studies have pointed to participatory surveillance as a promising alternative [5]. The active inclusion of communities through educational programs, workshops, and social mobilization has contributed to enhancing passive surveillance sensitivity, decentralising actions, and promoting shared responsibility in tackling the disease [6, 7].

Additionally, innovative solutions have been implemented in hard‐to‐reach areas, such as adapted traps and river‐based health units, which expand the reach of interventions without significantly increasing operational costs [8, 9]. Technological advances in spatial analysis, such as georeferencing tools and ecological niche modelling, have also improved the precision of risk area identification and guided more effective interventions [10].

Despite these advances, systematic analyses comparing the contributions of entomological surveillance across different endemic regions—considering their ecological, social, and operational particularities—remain scarce [9]. This gap justifies the need for studies that integrate these multiple dimensions to support more efficient and context‐specific public policies.

Thus, this study aims to investigate how entomological surveillance, through conventional and innovative strategies, has contributed to Chagas disease control in different regional contexts. The premise is that the effectiveness of this strategy depends not only on traditional methods but also on the integration of technological, social, and ecological approaches that account for the diverse realities of historically affected territories.

## 2 Methodology

This study is an integrative literature review with a high‐sensitivity search, guided by the following research question: ‘How does entomological surveillance contribute to Chagas disease control in different endemic regions?’ The PCC (Population, Concept, Context) strategy was used to formulate the question, defining:

- P (Population): Endemic regions
- C (Concept): Entomological surveillance
- C (Context): Chagas disease control

The search was conducted between December 2024 and February 2025 in the following databases: MEDLINE (PubMed), EMBASE (Elsevier), and the Virtual Health Library (VHL).

Inclusion criteria were: primary data scientific articles written in Portuguese, English, or Spanish. Exclusion criteria included articles unrelated to the research question and duplicates.

Descriptors were predefined based on controlled vocabularies: Medical Subject Headings (MeSH), Embase Subject Headings (Emtree), and Health Sciences Descriptors (DeCS). Terms included: ‘Entomology’, ‘Chagas Disease’, ‘*Trypanosoma cruzi* Infection’, ‘American Trypanosomiasis’, ‘Trypanosomiasis, American’, ‘Trypanosomiasis, South American’, and ‘South American Trypanosomiasis’.

Boolean operators were used for term combinations: OR for synonyms and AND to integrate different descriptors. No publication year restrictions or additional filters were applied to avoid excluding potentially relevant articles. Further details on the search strategy are available in the Supporting Information.

The search strategy combined controlled descriptors (MeSH, Emtree, DeCS) and their synonyms, integrated using the Boolean operators AND and OR, with no restriction on year of publication, and including articles in Portuguese, English, and Spanish; full details are provided in the Supporting Information.

Article selection and analysis were conducted using Rayyan Systems Inc., a web application that facilitated duplicate removal and screening based on predefined criteria. Three independent researchers performed a blinded initial review of titles, abstracts, and keywords (*n* = 567). A fourth researcher resolved selection conflicts to reach consensus on full‐text articles. After this stage, 79 full‐text articles were analysed, forming the final study corpus.

Original research articles with primary data on entomological surveillance of Chagas disease in endemic regions, available in full text in Portuguese, English, or Spanish, were included. Reviews, editorials, theses, dissertations, reports, protocols, commentaries, and studies unrelated to the guiding question or lacking relevant entomological data, as well as duplicates across databases, were excluded.

Data extraction was independently conducted by three reviewers using a standardised form based on the Ursi instrument. Discrepancies were resolved through consensus.

The extracted data were systematised in a standardised spreadsheet, including information on study context, vector species, surveillance approaches, and control strategies. Subsequently, a qualitative analysis was performed, with thematic categorisation of the articles into six analytical axes, and a descriptive analysis of the observed frequencies and trends (by species, geographic region, methodologies, and entomological indicators). The results are presented in thematic categories, with a narrative synthesis of the findings and illustrations in graphs and flowcharts.

A mixed‐methods approach was adopted, integrating qualitative thematic analysis of the contents with quantitative descriptors (species frequency, surveillance methods, types of control), which were presented through descriptive synthesis and graphical representations.

**FIGURE 1:**
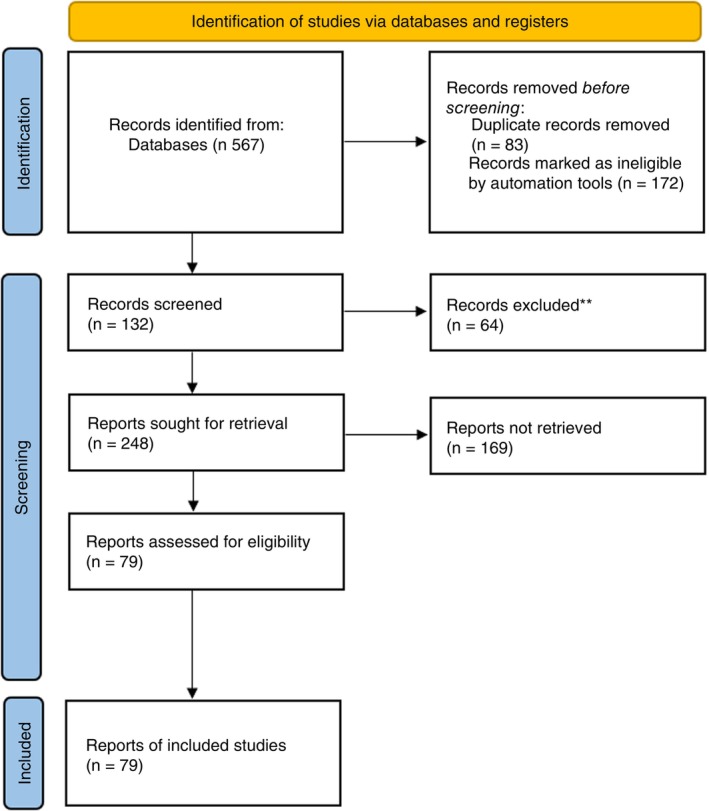
Flowchart of article selection. *Source:* Prepared by the authors (2025).

**FIGURE 2:**
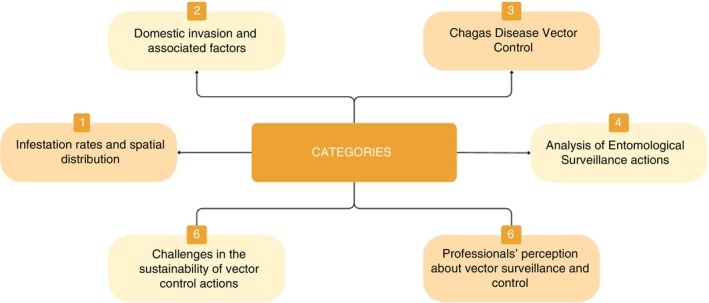
Categories. Study of category 1: (1, 2, 3, 4, 5, 6, 11, 12, 13, 14, 15, 16, 17, 20, 21, 22, 24, 25, 26, 27, 28, 29, 36, 38, 39, 41, 42,43, 45,46, 49, 50, 51, 55, 72); Estudos da categoria 2: (29, 30, 31, 32, 34, 36, 37, 38, 39, 40, 41, 42, 43, 44, 45, 46, 50, 54, 55, 56, 57, 58, 59, 60, 61, 62, 63, 64, 65, 66, 68, 69, 71,72, 77, 73); Estudos da categoria 3: (1, 4, 5, 6, 7, 9, 11, 12, 13, 14, 15, 19, 20, 22, 26, 28, 29, 30, 31, 32, 33, 35, 36, 37, 38, 39, 40, 41, 42, 43, 44, 45, 46, 47, 48, 49, 50, 52, 53, 55, 58, 59, 60, 61, 62, 63, 65, 66, 67, 68, 69, 71, 73). Estudos da categoria 4: (1, 2, 4, 5, 6, 7, 8, 9, 10, 11, 12, 13, 14, 15, 16, 17, 18, 19, 20, 21, 22, 23, 24, 25, 26, 27, 28, 29, 32, 33, 35, 36, 37, 39, 42, 47, 48, 49, 52, 55,57,58, 59, 60, 64, 65, 66, 68, 71). Estudos da categoria 5: (1, 2, 3, 4, 5, 6, 7, 8, 9, 10, 11, 12, 13, 14, 15, 16, 17, 18, 19, 20, 21, 25, 26, 27, 32, 34, 36, 37, 39, 43, 49, 52, 53, 54, 55, 56, 57, 58, 59, 60, 61, 65, 66, 68, 70). Estudos da categoria 6: (1, 2, 3,4, 5, 6, 7, 8, 9, 10, 11, 12, 13, 14, 15, 16, 17, 18, 19, 20, 21, 22, 23, 24, 25, 26, 27, 28, 29, 30, 31, 32, 33, 34, 35, 36, 37, 38, 39, 40, 41, 42, 43, 44, 45, 46, 48, 49, 50, 51, 52, 53, 54, 55, 56, 57, 58, 59, 60, 61, 62, 63, 64, 65, 66, 67, 68, 69, 70, 71, 72, 73, 74, 75, 76, 77, 78, 79). *Source:* Prepared by the authors (2025).

Figure 1 presents a flowchart illustrating the identification, screening, eligibility, and inclusion process. Data were structured into six categories to discuss study findings (Figure 2, created using Canva).

The analytical categories (Figure 2) were constructed inductively, based on the full‐text reading of the included articles. Two researchers performed the initial coding of the texts, identifying recurring terms, stated study objectives and main analytical foci (e.g., entomological indices, household invasion and control strategies). The codes were then grouped into related thematic axes, resulting in six final categories defined by consensus among the reviewers.

Ethical approval was not required, as this study used only secondary data from published articles.

## 3 Results

The study provided a detailed overview of how entomological surveillance contributes to the control of Chagas disease across different endemic regions.

The selection of the six categories resulted from a thematic analysis of the 79 articles included in the review, aiming to reflect the most recurrent and relevant axes in the literature on entomological surveillance for Chagas disease. This classification was chosen because it encompasses essential technical, social, and operational dimensions needed to understand the complexity of vector control. Thus, entomological indices and spatial mapping were highlighted for representing classical indicators of infestation and colonization, coupled with the use of geotechnologies and geographic information systems for risk stratification. Factors associated with household invasion were included for demonstrating the influence of environmental, structural, and demographic aspects on the domiciliation of sylvatic species, broadening the understanding of the transmission process. Vector control strategies formed a distinct category due to the diversity of measures identified, ranging from chemical control with insecticides to innovative techniques such as traps, citizen science, educational actions, and housing improvements. Surveillance methods were grouped to encompass both traditional approaches, such as active search and the man–hour technique, and innovations like predictive models and participatory surveillance. Professional perceptions were deemed fundamental, as they revealed the practical perspective of endemic disease agents, their field constraints, and the importance of continuous training for effective action. Finally, challenges to the sustainability of interventions synthesized the barriers reported in the literature, including discontinuity of public policies, insecticide resistance, and persistent peridomestic reinfestation. In this way, the six‐axis categorisation ensured a systematic organisation of findings, enabling a critical, comparable, and comprehensive analysis of the diverse realities of entomological surveillance in endemic regions.

Although peridomestic infestation is partially captured within the broader category of ‘Entomological indices and spatial distribution’, the analysis of the 79 studies showed that peridomestic structures (such as chicken coops, corrals, wood piles and other annexes) consistently emerge as critical and persistent foci of triatomine colonization. For this reason, peridomestic infestation was examined as a specific analytical dimension, given its central role as a reservoir for reinfestation of human dwellings and as a major operational challenge for vector control programs.

### 3.1 Category 1: Infestation Indices and Spatial Distribution

The reviewed studies show that triatomine infestation is quantified using various entomological indicators. These metrics enable understanding of vector presence intensity and persistence in endemic areas. The main indices include household infestation rate, vector density, and colonization rate—key measures for characterising local risk. Additionally, spatial distribution of triatomines is increasingly depicted through thematic maps that help identify priority intervention zones. These representations are often coupled with geotechnologies and Geographic Information Systems (GIS), allowing integrated analysis of entomological, environmental, and territorial data. Such integration has proven essential for enhancing surveillance capacity and guiding more efficient, context‐sensitive control strategies. Overall, the analytical tools employed in entomological studies revolve around three axes: (1) calculation of entomological indices; (2) mapping of vector spatial distribution; and (3) use of geotechnologies and/or GIS. When used in concert, these approaches enrich data interpretation and strengthen public health responses to persistent vector transmission of Chagas disease.

### 3.2 Category 2: Household Invasion and Associated Factors

Regarding category 2, household invasion by triatomines is associated with environmental and demographic factors that facilitate vector domiciliation. Environmental factors include climate, vegetation, and urbanisation processes; demographic factors include population density and housing conditions. These aspects contribute to triatomine adaptation to domestic habitats, increasing the infestation risk and, consequently, transmission of Chagas disease. Evidence of wild species such as *Triatoma brasiliensis and T. pseudomaculata* in domestic settings underscores the importance of continuous entomological surveillance [11]. The presence of these species indoors highlights their capacity for domiciliation and reinforces the need for vigilance in endemic regions. The analysis of these factors underscores infestation complexity and the importance of monitoring for disease control.

### 3.3 Category 3: Vector Control of Chagas Disease

**FIGURE 3:**
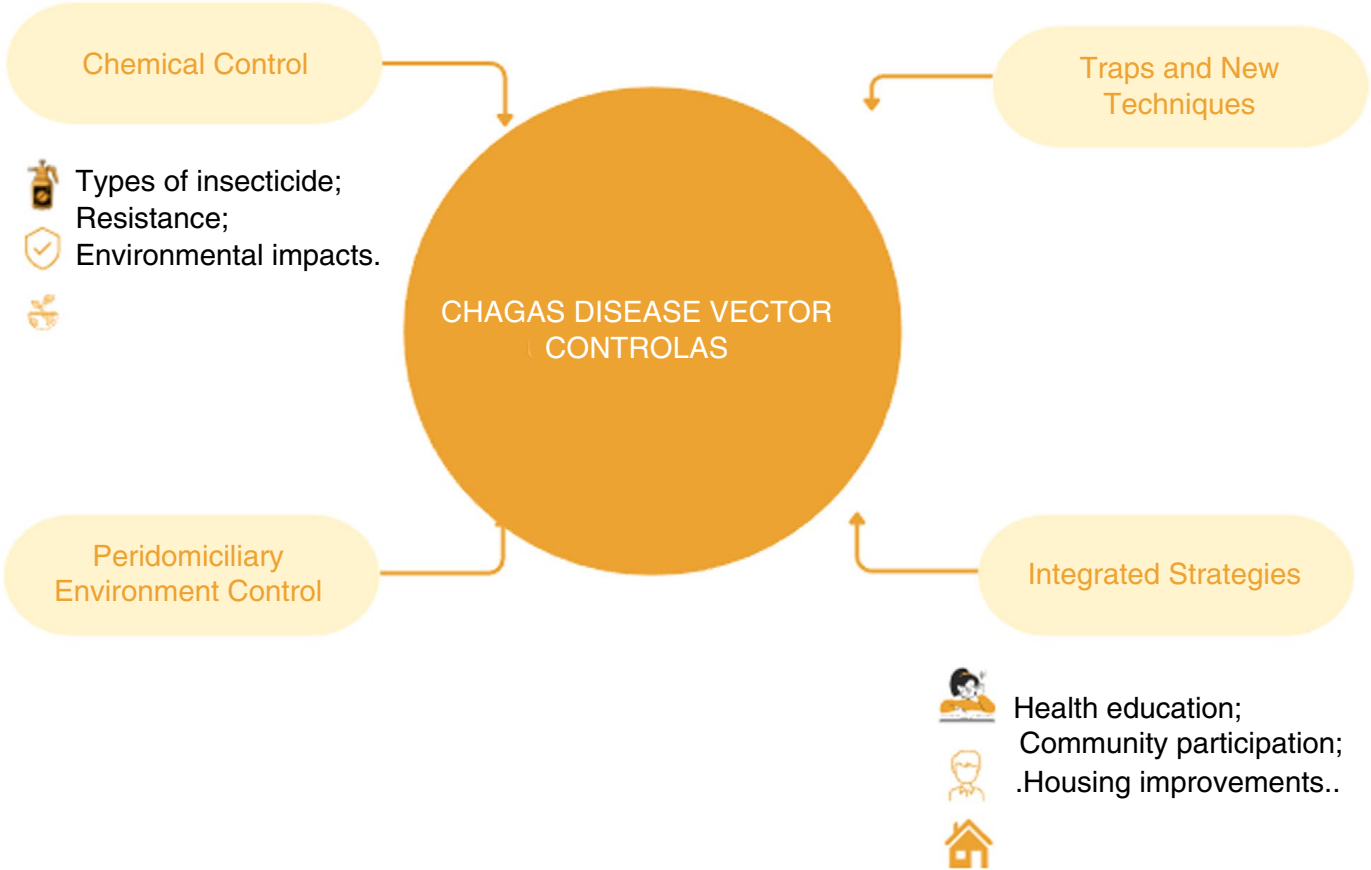
Schematic representation of vector control approaches for Chagas disease carried out by entomological surveillance. Controle Químico: (1, 3, 4, 5, 6, 7, 10, 11, 14, 15, 20, 21, 22, 24, 26, 27, 30, 36, 39, 41, 43, 45, 42, 52, 55, 56, 57, 58, 61, 62, 63, 64, 65, 66, 67, 72, 73, 74, 75, 76, 77, 78, 79); Armadilhas e Novas Técnicas: (2, 3, 6, 8, 10, 12, 13, 17, 18, 21, 23, 28, 29, 30, 32, 33, 35, 37, 38, 39, 42, 43, 44, 47, 48, 49, 55, 56, 57, 58, 61, 62, 63, 64, 65, 66, 67, 72, 73, 74, 75, 76, 77, 78, 79); Controle no Ambiente Peridomiciliar: (1, 2, 3, 4, 5, 6, 7, 8, 9, 10, 11, 12, 13, 14, 15, 16, 17, 18, 19, 20, 21, 22, 23, 24, 25, 26, 27, 29, 31, 32, 33, 35, 36, 37, 38, 39, 41, 42, 43, 44, 45, 46, 48, 49, 52, 53, 55, 56, 57, 58, 59, 60, 61, 62, 63, 64, 65, 66, 67, 68, 69, 70, 71, 72, 74, 75, 76, 78, 79); Estratégias Integradas: (1, 4, 5, 6, 7, 9, 11, 12, 13, 14, 15, 19, 20, 22, 26, 31, 32, 36, 37, 40, 43, 45, 46, 49, 50, 52, 53, 54, 55, 56, 57, 58, 59, 60, 61, 62, 63, 64, 65, 66, 67, 68, 69, 70 a 79). *Source:* Prepared by the authors (2025).

Category 3 covers studies on vector control strategies, grouped into four axes—presented in Figure 3: chemical control (insecticide types, resistance and environmental impacts); traps and new surveillance techniques; peridomestic environmental actions; and integrated strategies such as health education and housing improvements. The reviewed articles examine the historical evolution of measures, operational challenges, and methodological innovations aimed at reducing triatomine infestation and preventing *Trypanosoma cruzi* vector transmission.

Chemical control: Frequent use of pyrethroids, challenges related to vector resistance, and persistent reinfestation despite spraying campaigns.

Traps and new techniques: Use of adhesive traps, ecological modelling, and citizen science to complement passive surveillance.

Peridomestic measures: Peridomestic structures such as corrals, chicken coops, and debris are identified as primary colonization sites.

Integrated strategies: Health education, community participation, and housing improvements are essential for the long‐term sustainability of vector control efforts.

This integrated analysis reveals the complexity of vector dynamics and the need for multicomponent, ecologically and socially adapted approaches.

### 3.4 Category 4: Analysis of Entomological Surveillance Activities

**FIGURE 4:**
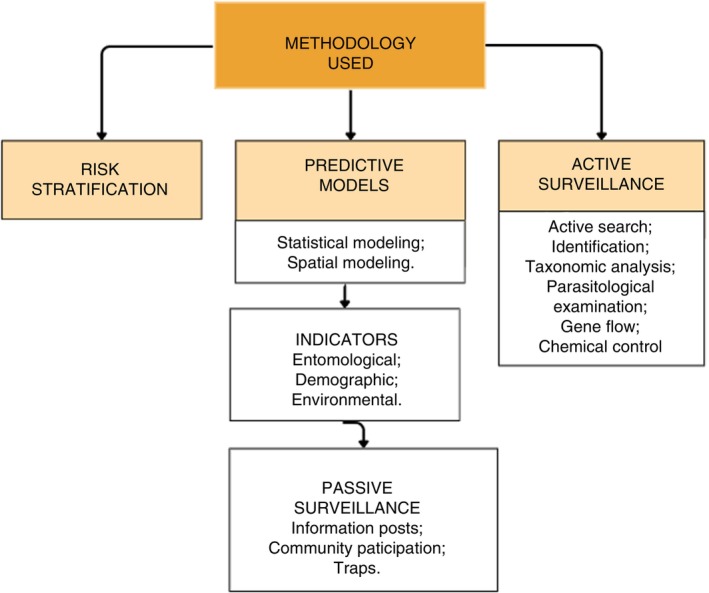
Schematic representation of the methodologies used by entomological surveillance for the control of Chagas disease. Estratificação de risco: (1, 4, 11, 13, 14, 15, 42, 55,57,59, 60, 64, 65, 66, 68, 71); Modelos preditivos/indicadores/vigilância passiva: (1, 2, 4, 5, 6, 7, 8, 9, 10, 11, 12, 13, 14, 15, 16, 17, 18, 19, 20, 21, 24, 25, 26, 27, 55, 57,59, 60, 64, 65, 66, 68, 67); Vigilância Ativa: (1, 2, 4, 5, 9, 10, 11, 12, 13, 14, 15, 16, 17, 18, 19, 20, 21, 22, 23, 24, 25, 26, 27, 28, 29, 32, 33, 35, 36, 37, 39, 47, 48, 49, 52, 55, 58, 59, 60, 64, 65, 66, 68, 71). *Source:* Prepared by the authors (2025).

Entomological surveillance actions are fundamental to controlling Chagas disease, especially given residual infestation and household reinvasion. Studies in this category highlight the diversity of approaches used by surveillance programs, ranging from traditional active search methods to advanced predictive risk mapping. Key methods include territorial stratification based on entomological and environmental indicators, predictive modelling, standardised active searches (e.g., man‐hour technique), parasitological examinations, and innovative tools like adhesive traps and citizen science. These findings were systematised in Figure 4, presenting the main methodologies employed in entomological surveillance.

### 3.5 Category 5: Professionals' Perceptions of Surveillance and Vector Control

This category explores the perceptions of professionals directly involved in surveillance, focusing on endemic agents' experiences. Literature shows that agent engagement in detection, monitoring, and vector control—both in urban and rural areas—leads to better outcomes when continuous training is provided. However, their work is hindered especially in municipalities with decentralisation and scarce resources. While ongoing professional training is essential, the lack of transport, educational materials, technical support, and structured training impedes agents' effectiveness. Understanding these professionals' perspectives provides deep insight into operational limitations and points to avenues for strengthening control actions.

### 3.6 Category 6: Challenges to Sustainability of Vector Control Actions

**FIGURE 5:**
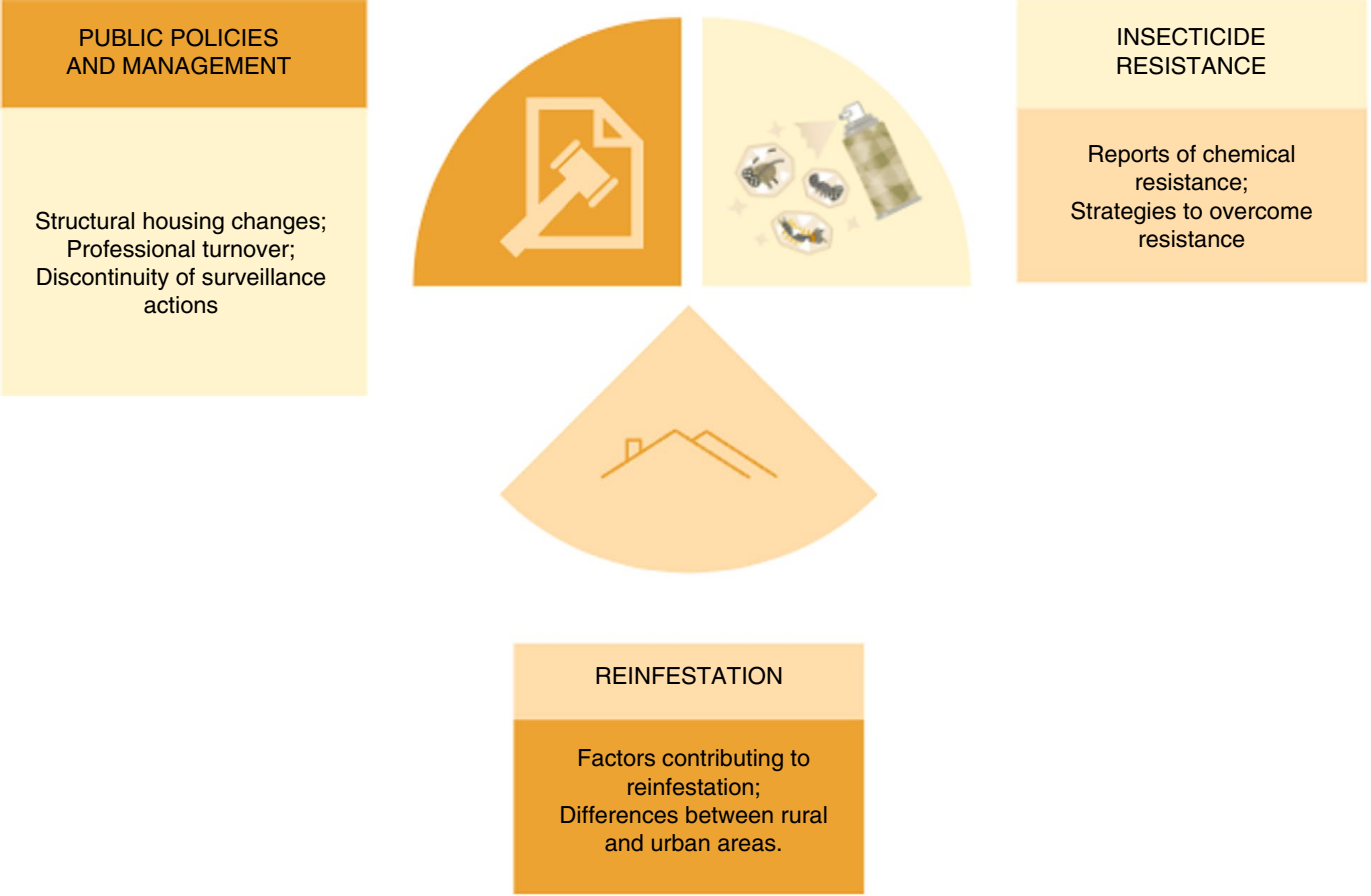
Schematic representation of the challenges for the sustainability of Chagas disease control actions. Políticas públicas e gerenciamento: (1, 2, 4, 7, 9, 10, 11, 13, 14, 15, 16, 17, 18, 19, 20, 22, 23, 24, 25, 26, 27, 31, 34, 35, 37, 38, 39, 41, 42, 43, 44, 45, 46, 49, 51, 52, 53, 55, 56, 57, 58, 63, 65, 72, 73); Reinfestação: (1, 2, 3, 4, 5, 6, 7, 8, 9, 10, 11, 12, 13, 14, 15, 16, 17, 18, 19, 20, 21, 22, 23, 24, 25, 26, 27, 28, 29, 30, 31,32, 33, 34, 35, 36, 37, 38, 39, 40, 41, 42, 43, 44, 45, 46, 48, 49, 50, 53, 54, 55, 56, 57, 58, 59, 60, 61, 62, 63, 64, 65, 66, 67, 68, 69, 70, 71, 72, 73, 74, 75, 76, 77, 78, 79); Resistência a inseticidas: (4, 10, 14, 20, 22, 23, 24, 53, 55, 56, 57, 58, 63, 68, 71, 72). *Source:* Prepared by the authors (2025).

Sustaining vector control efforts faces structural, operational, and ecological challenges that compromise long‐term entomological surveillance. This category synthesizes the main barriers into three interdependent axes, as shown in Figure 5: (1) weaknesses in public policies and local management (e.g., discontinuity in actions, staff turnover, territorial structural changes); (2) chemical resistance of vectors to applied insecticides, reducing spray efficacy; and (3) ongoing household reinfestation driven by environmental, behavioural, and structural factors, with notable differences in rural versus urban areas. These elements underscore the need for territorialized, resilient vector control integrated with health, housing, and environmental policies.

Figure 3 shows that chemical control with pyrethroids remains the most frequently reported strategy in the studies, although the literature highlights limitations related to vector resistance and low residual effect in peridomestic structures. In contrast, integrated interventions that combine housing improvements, environmental management of the peridomicile and educational actions, although less numerous, tend to yield more sustainable results in reducing triatomine infestation and reinfestation.

As summarized in Figure 4, conventional methods of active search and standardised entomological indices still predominate in the studies analysed. However, approaches that incorporate geotechnologies, ecological modelling, and citizen science, although less frequent, stand out for increasing the sensitivity of surveillance and targeting control actions more precisely.

## 4 Discussion

When analysing the six categories together, it becomes clear that the studies are not limited to isolated dimensions but reveal important intersections. The entomological indices and spatial mapping (Category 1) directly interact with the environmental and demographic factors associated with household invasion (Category 2), since risk maps only gain accuracy when variables such as type of housing, presence of animals, and land use are incorporated. These elements, in turn, largely explain the challenges of reinfestation and sustainability (Category 6), reinforcing that territorial vulnerability cannot be overcome solely through chemical campaigns. In this sense, studies on control strategies (Category 3) and professionals' perceptions (Category 5) converge in emphasising that insecticide spraying, although central, is insufficient without housing improvements and community engagement. There is also complementarity between surveillance methods (Category 4) and citizen science, which have proven effective in contexts of limited institutional coverage but whose effectiveness depends on the social context, as shown by contradictions observed in experiences from Brazil and Venezuela. Finally, some divergences emerge in the literature: while certain studies report satisfactory residual effects of pyrethroids, others reveal systematic failures associated with vector resistance and the complexity of peridomestic ecotopes. This interplay of reinforcements, complementarities, and contradictions demonstrates that entomological surveillance only achieves effectiveness when analysed in an integrated manner, overcoming thematic fragmentation.

Entomological surveillance is an indispensable and strategic component in combating Chagas disease, particularly given the ecological and operational complexities of vector control in endemic regions. Empirical studies in Latin America—especially Mexico, Argentina, Ecuador, and Brazil—report high intra‐ and peridomestic infestation rates by *Triatoma dimidiata* , *T. infestans* , *Rhodnius ecuadoriensis* , and other priority vectors [12, 13, 14, 15].

Several empirical investigations conducted in Latin American territories—notably in Mexico, Argentina, Ecuador and Brazil—report high intra‐ and peridomestic infestation rates by key vector species [11, 13, 14, 15, 16, 17].

These findings underscore the importance of entomological indices in epidemiological characterisation, especially when paired with diagnostic‐enhancing tools like artificial sensors and phenolphthalein faecal tests [13, 14].

Spatial analysis via GIS has proved essential for identifying critical zones by overlaying environmental, structural, and socioeconomic layers [5, 18]. The use of predictive models—such as ecological niche models and metapopulation simulations [19]—has enabled robust understanding of vector dynamics and latent risk areas, even in places once considered controlled.

Despite the central role of insecticide‐based interventions, substantial limitations exist regarding efficacy, durability, and reinfestation prevention. Specific pyrethroid formulations retained residual activity [20], but reinvasion in peridomestic ecotopes persisted after multiple applications [21, 22]. These issues highlight the need for permanent structural and environmental measures, such as housing improvement and ecohealth approaches [23].

Peridomestic settings—chicken coops, corrals, debris—remain persistent vector reservoirs, prompting the need for environmental management [24, 25]. Conventional methods are often inadequate, necessitating alternatives like semiochemical traps, radiofrequency devices, and sniffer dogs [26, 27]. Additionally, molecular and genetic analyses are valuable complementary tools, especially for characterising hard‐to‐reach wild foci [28].

Participatory technologies in entomological surveillance are increasingly important. Digital tools and participatory methodologies support continuous data collection. Citizen science platforms integrated with georeferencing systems enable real‐time vector mapping, promoting territory‐based, responsive, and socially engaged surveillance [29, 30]. This is particularly relevant in areas with limited programmatic coverage, where community engagement becomes a strategic asset.

Innovative traps baited with semiochemicals (hexanal, octanal) have shown high sensitivity in detecting triatomines, outperforming conventional manual methods in low‐density settings (Gürtler et al., 2012). Combined with faecal sensors and artificial refuge devices, they extend surveillance beyond manual inspection.

Digital tools such as vector‐recognition algorithms [31] and geotechnologies ([32]; Gürtler et al., 2005) enhance integrated surveillance, enabling hotspot detection and precise delimitation of priority areas.

From an ecological standpoint, forest fragmentation, land‐use change, and synanthropic animals favour vector migration from wild to anthropogenic habitats (Ferro; [2, 5]). Even high‐end residences built on formerly forested land may harbour infected vectors, showing that changes in land use do not eliminate but rather perpetuate entomological risks (Nascimento et al., 2013).

The domiciliation and reinfestation process is multifactorial, shaped by environmental degradation, housing conditions, and vector adaptability [33, 34, 35]. Studies show vector presence in urban and periurban areas previously deemed low‐risk [20, 27, 33, 34, 35].

Housing structures—such as cracked walls, thatched roofs, and annexes like chicken coops and pigsties—are strongly associated with persistent vectors [25, 36], providing refuge, food, and breeding sites even after repeated insecticide treatments [20, 21].

Different vector species vary in domiciliation tendency: *T. infestans* and *T. dimidiata* show strong intradomiciliary colonization (Hernández et al., 2010; Becerril et al., 2010), whereas *T. barberi* , *T. vitticeps* , *T. maculata* , and *T. pallidipennis* display partial domiciliation or repeated invasive behaviour (Ferreira et al., 2005; [8, 18]). The presence of eggs and nymphs—especially in eucalyptus palms and wooden structures—suggests peridomestic reproductive cycles [17].

Spatial and population studies [19, 28] show that connectivity between wild and domestic habitats and gene flow between sylvatic and domiciliated populations undermine the effectiveness of control solely based on indoor spraying.

Demographic factors and host management are also critical. The presence of dogs and chickens near dwellings increases vector‐human contact and modulates seasonal transmission risk [37, 38]. Surveillance must adopt an integrated approach that considers spatial analysis, vector ecology, housing type, animal management, and land use.

From this perspective, vector domiciliation is an adaptive, progressive, and multifactorial process. Species like *T. infestans* , *T. dimidiata* , and *T. barberi* have high colonization potential ([15]; Becerril et al., 2010), while others like *T. maculata* and *T. vitticeps* remain predominantly peridomestic with potential to invade dwellings (Ferreira et al., 2005; [8]).

### 4.1 Control Strategies

Although widely used, pyrethroids such as deltamethrin and cypermethrin present significant limitations—especially on exposed peridomestic structures—due to factors like weather exposure and ecological complexity [20, 39]. Insecticide resistance and inefficacy in complex habitats compromise residual and sustained control [27].

Triatomines persist particularly in environments like chicken coops, corrals, and woodpiles, which serve as recurrent reinfestation foci [40, 41], emphasizing the need for environmental management and physical restructuring of peridomestic surroundings.

### 4.2 Integration and Community Participation

The integration of innovative surveillance methods, community engagement, and structural strategies is regarded as the most promising approach. Campaigns such as ‘#TraeTuChipo’ [29] and participatory models described by Weeks et al. [42] and Silva et al. [43] illustrate how decentralised surveillance with community involvement and local agent training enhances early detection and institutional response.

Surveillance effectiveness is evaluated through operational and epidemiological indicators like household positivity, *T. cruzi* infection rate, time to recolonization, and notification response. Studies [34, 37, 44] confirm that beyond initial reductions, sustained results depend on continuous monitoring and locally tailored strategies.

Entomological surveillance effectiveness in controlling Chagas disease is closely linked to well‐trained endemic agents (ACEs), active community participation, and strategies adapted to ecological and socio‐environmental conditions [40]. Research [45] shows that health professionals' perception and engagement significantly vary depending on institutional conditions and epidemiological vulnerability.

In Rio Grande do Norte, Brazil, agents emphasized active surveillance for early detection of *T. brasiliensis* and *T. pseudomaculata* , even in areas with routine control interventions [16]. In Loja province, Ecuador, the absence of systematic surveillance programs led to low recognition among professionals and the population of the link between triatomines and *T. cruzi* transmission [17].

Studies also underscore structural vulnerabilities that hinder ACEs' performance. Souza et al. [46] point to high staff turnover, lack of continuous training, and poor integration with primary health care services in Minas Gerais. Villela et al. [45] and Priotto et al. [40] report that lack of material resources and regular technical training compromises sustainability, particularly in regions with low spontaneous vector notification.

Conversely, innovative, community‐based experiences show promise. In Comapa, Guatemala, Castro‐Arroyave et al. [23] documented sustainable, culturally adapted interventions involving residents and professionals from health, engineering, and social sciences—positively impacting both surveillance and community organisation. In Brazil, Silva et al. [43] and Barbosa et al. [34] emphasize that decentralised, socially mobilised surveillance action remains effective even after elimination of *T. infestans* .

Technical‐operational qualification of ACEs is also critical. Cecere et al. [20] and Rodríguez‐Planes et al. [19] showed that selective insecticide application and risk‐focused ecotope identification (e.g., chicken coops, corrals) require specific technical knowledge plus planning aligned with seasonal vector biology [44]. Thus, chemical control effectiveness depends on field expertise and adaptive response capacity.

In contexts with institutional fragility, citizen science‐based strategies have proven effective. For example, Venezuela's ‘#TraeTuChipo’ campaign [29] mobilised the public via digital platforms to identify vectors and *T. cruzi* infections, even amid health service collapse.

The evidence shows that successful Chagas surveillance and control hinges on combining standardised entomological metrics, geospatial information technologies, predictive ecological modelling, and participatory community strategies to optimise resource allocation and ensure long‐term effectiveness across different epidemiological settings. Effective action requires continued technical training, adequate operational infrastructure, community engagement, and intersectoral integration among surveillance, primary health care, and health education.

This study has several limitations that should be considered when interpreting the findings. The primary limitation concerns the methodological heterogeneity among the included articles and the scarcity of standardised data on the long‐term effectiveness of different surveillance strategies. There is also a predominance of studies from Brazil, Argentina, and Mexico, which may limit the generalizability of the results to other, less‐represented endemic settings. Additionally, variability in the entomological indicators used across studies hampers direct comparisons. As an integrative review, the study is also susceptible to publication bias, since studies reporting negative or inconclusive results are less likely to be available in indexed databases. Despite these constraints, by integrating diverse methodological and contextual approaches, this review provides a critical and comprehensive synthesis of the contributions of entomological surveillance to Chagas disease control, with potential to inform more context‐sensitive and sustainable public policies.

Regarding the geographical distribution of the evidence, most of the included studies were conducted in Brazil, Argentina, and Mexico. Although we initially mentioned this as a potential limitation for generalising the findings to other endemic countries, this pattern may also reflect the larger territorial extension, greater epidemiological heterogeneity, and higher diversity of triatomine species in these three countries, as well as their longer tradition of research and surveillance programmes. In this sense, the concentration of publications does not necessarily bias the conclusions, but rather highlights where entomological surveillance has been most extensively documented.

In this review, the country of origin of each study was recorded and summarized descriptively, but no formal meta‐analytical or inferential comparison between countries was performed. Therefore, the predominance of Brazil, Argentina, and Mexico was treated as a descriptive feature of the evidence base rather than a source of systematic bias.

## 5 Conclusion

After a detailed analysis and categorisation of the 79 articles, this review shows that entomological surveillance for Chagas disease has evolved from predominantly manual and chemically centered strategies to more integrated approaches that combine classical indices, spatial analysis tools, and community participation. Emerging technologies, such as geotechnologies, ecological modelling, and digital platforms for citizen science, are reshaping surveillance by increasing sensitivity, refining risk stratification, and enabling more timely and territorialized responses.

Despite these advances, a major challenge that emerges from the evidence is the ecological and operational impact of the replacement of historically predominant vectors by secondary species, which exert continuous pressure on households and peridomestic environments. This process tends to be intensified by deforestation, land‐use changes and global warming, which favour the intrusion and adaptation of sylvatic triatomines into human‐modified habitats, demanding surveillance models that are more resilient, adaptive and environmentally informed. In this context, the future of entomological surveillance will depend on the capacity to integrate technological innovation, environmental and climate perspectives and sustained community engagement into long‐term, multisectoral control strategies.

## Conflicts of Interest

The authors declare no conflicts of interest.

## Supporting information


**Data S1:** PRISMA 2020 flow diagram for new systematic reviews which included searches of databases and registers only.

## Data Availability

The data that support the findings of this study are available on request from the corresponding author. The data are not publicly available due to privacy or ethical restrictions.
