# Supplementary material for: The Contribution of Entomological Surveillance to the Control of Chagas Disease in Endemic Regions: An Integrative Literature Review
Source: Trop Med Int Health. 2026 Apr 22;31(7):832–42. doi: 10.1111/tmi.70128 (PMC13331536; doi:10.1111/tmi.70128)
Supplement: Supplementary file 1 — Data S1: PRISMA 2020 flow diagram for new systematic reviews which included searches of databases and registers only. [file TMI-31-832-s001.docx]

**Identification of studies via databases and registers**

Records removed *before screening*:

Duplicate records removed (n = 83 )

Records marked as ineligible by automation tools (n = 172)

Records identified from*:

Databases (n 567 )

**Identification**

Records screened

(n = 312)

Records excluded**

(n = 64)

Reports sought for retrieval

(n = 248)

Reports not retrieved

(n = 169 )

**Screening**

Reports assessed for eligibility

(n = 79)

Reports of included studies

(n = 79)

**Included**


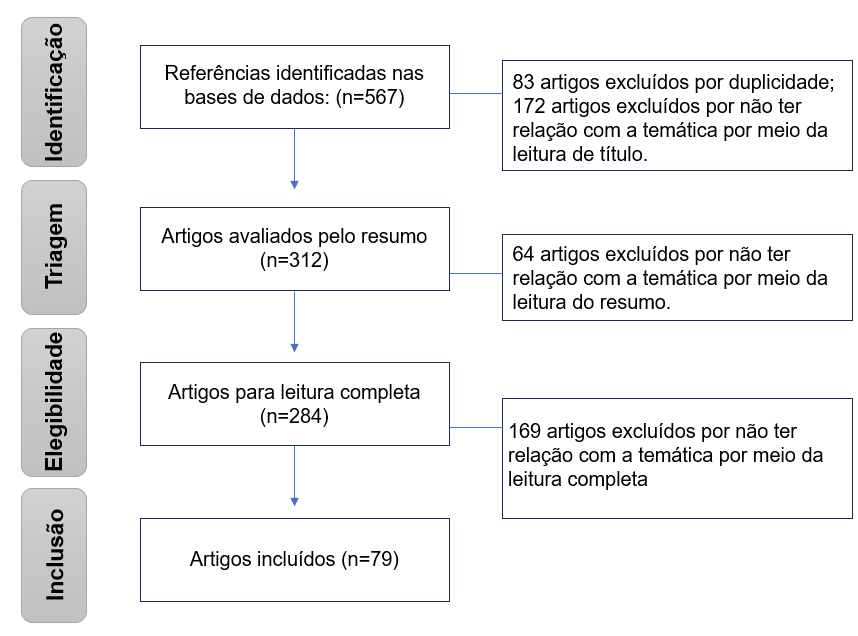


*Consider, if feasible to do so, reporting the number of records identified from each database or register searched (rather than the total number across all databases/registers).

**If automation tools were used, indicate how many records were excluded by a human and how many were excluded by automation tools.

*From:*  Page MJ, McKenzie JE, Bossuyt PM, Boutron I, Hoffmann TC, Mulrow CD, et al. The PRISMA 2020 statement: an updated guideline for reporting systematic reviews. BMJ 2021;372:n71. doi: 10.1136/bmj.n71
